# Relationship between Objectively Measured Transportation Behaviors and Health Characteristics in Older Adults

**DOI:** 10.3390/ijerph121113923

**Published:** 2015-10-30

**Authors:** Michelle Takemoto, Jordan A. Carlson, Kevin Moran, Suneeta Godbole, Katie Crist, Jacqueline Kerr

**Affiliations:** 1 Department of Family Medicine Public Health, University of California, San Diego, CA 92093, USA; E-Mails: sgodbole@ucsd.edu (S.G.); kcrist@ucsd.edu (K.C.); jkerr@ucsd.edu (J.K.); 2 Center for Children’s Healthy Lifestyles and Nutrition, Children’s Mercy Hospital, Kansas City, MO 60148, USA; E-Mail: jacarlson@cmh.edu; 3 Department of Preventive Medicine, Northwestern University, Chicago, IL 60611, USA; E-Mail: Kevin.Moran@northwestern.edu

**Keywords:** physical mobility, life-space mobility, older adults, Global Positioning System (GPS), physical functioning, psychological functioning, cognitive functioning, health

## Abstract

This study used objective Global Positioning Systems (GPS) to investigate the relationship between pedestrian and vehicle trips to physical, cognitive, and psychological functioning in older adults living in retirement communities. Older adults (N = 279; mean age = 83 ± 6 years) wore a GPS and accelerometer for 6 days. Participants completed standard health measures. The Personal Activity and Location Measurement System (PALMS) was used to calculate the average daily number of trips, distance, and minutes traveled for pedestrian and vehicle trips from the combined GPS and accelerometer data. Linear mixed effects regression models explored relationships between these transportation variables and *physical, psychological* and *cognitive functioning*. Number, distance, and minutes of pedestrian trips were positively associated with physical and psychological functioning but not cognitive functioning. Number of vehicle trips was negatively associated with fear of falls; there were no other associations between the vehicle trip variables and functioning. Vehicle travel did not appear to be related to functioning in older adults in retirement communities except that fear of falling was related to number of vehicle trips. Pedestrian trips had moderate associations with multiple physical and psychological functioning measures, supporting a link between walking and many aspects of health in older adults.

## 1. Introduction

There is a well-documented trend in the increasing number of adults over the age of 65 in the United States and worldwide. According to the Administration on Aging (AOA), the number of adults over 65 is expected to grow from 39.6 million in 2009 to about 72.1 million by 2030, representing 19% of the population [1]. Not only are more people turning 65 each day, but people are also living longer than ever before [2,3,4]. Many older adults wish to remain in their homes, but studies have shown that environments in retirement communities can also support healthy activities [5]. It is important to understand the impact of place on continued mobility in older adults.

As individuals age, there is a reduction in mobility that has been linked to a number of health outcomes [6]. Mobility can be defined as physical mobility, which is highly related to the ability to walk and includes an individual’s capability to engage in activities of daily living [7]. Several studies have indicated that physical mobility is an important element in quality of life as it allows older adults to continue leading dynamic and independent lives [8,9]. Physical mobility delays the onset of disabilities, postpones frailty, and contributes to subjective well-being and life satisfaction [6].

Mobility can also be defined as movement extending from within one’s home to movement beyond one’s town or geographic location which is known as life-space mobility [10]. Life-space mobility has been linked to physical function with mobility in one’s home and community surroundings highlighted as strong predictors of physical disability [11]. The degree of an older adult’s life-space mobility has also been found to correlate with his/her social and emotional life. For example, people who are more mobile report less loneliness and stronger feelings of community integration [8]. In older persons with cognitive decline, maintaining out-of-home life-space mobility is important as it has been found to positively impact their enjoyment, feelings of integration, satisfaction, and sense of autonomy and identity [12,13]. For the majority of Americans, driving is important for maintaining quality of life and independence [13]. Driving cessation is also associated with a number of negative consequences including increased social isolation, depression, and a greater risk of long-term care placement [13].

The relationship between physical and life-space mobility is especially important given the increasing number of older adults. Most previous studies that have explored the relationship between physical mobility and health in an older adult population have relied on measures of functional independence including self-report measures of an individual’s ability to participate in activities of daily living including bathing or dressing [7]. Other methods have included objective measures of physical functioning such as gait [14,15]. The most common measure of life-space mobility is the self-reported Life-Space Assessment, which measures mobility based on the distance a participant reports moving during the 4 weeks preceding the assessment [16]. Other measurement methods involve travel journals, which ask participants to record a location and travel behavior for 12 h at 5 min intervals [13]. This type of data collection can be extremely burdensome and, given the memory impairments associated with aging [17], the validity of these self-report measures may be low [18,19].

New measurement methods have been deployed to reduce the burden on the participant and to capture more accurate data based on objective measures. One such method involves equipping participants with Global Positioning System (GPS) tracking devices. These devices capture real-time data that can objectively track the location of an individual [20]. Additionally, GPS technology overcomes a number of limitations associated with previous assessment methods (*i.e.*, travel diaries, self-report surveys) by reducing potential for social desirability and reporting fatigue because the device passively captures data without burdening the participant. Further it has been found that participants round up travel times and are very poor estimators of travel distance [21]. These issues are likely magnified in older adults. The data from these devices can be processed and analyzed to detect a variety of transportation variables including trips, locations, mode of transportation, and whether a participant is indoors or outdoors [20,22,23]. These variables can be especially valuable when trying to identify certain patterns related to specific populations of interest. Previous studies that have used GPS data have involved different population groups such as individuals with Alzheimer’s disease or cognitive impairment [24,25]. Additionally, studies have used GPS devices to capture mobility related behaviors (e.g., activity space, out of home mobility, and life-space) [26,27,28]. Studies have yet to relate GPS transportation variables to multiple health characteristics. Therefore, the purpose of the current study was to explore the utility of GPS data to explore transportation behaviors as related to various health measures in a population of healthy older adults residing in retirement communities. Specifically, we evaluated the relation of the (1) number of pedestrian and vehicle trips per day; (2) distance traveled per day in pedestrian and vehicle trips; and (3) daily minutes spent in pedestrian and vehicle trips, to physical, psychological, and cognitive functioning.

## 2. Methods

### 2.1. Study Design and Procedures

Data for this study were from baseline assessments in the Multilevel Intervention for Physical Activity in Retirement Communities (MIPARC) which was a group randomized controlled trial consisting of adults (n = 279) over 65 years of age residing in retirement communities throughout San Diego County [29]. Twenty sites that were identified from the county Elder Care database met the criteria for participation which included: more than 100 residents, independent living accommodations, and a park or shops within walking distance (*i.e*., one mile). A total of 11 retirement communities completed the study. Additional details regarding the study design are described elsewhere [29].

### 2.2. Study Participants

Eligible participants completed an informed consent process and had to meet the following criteria to participate in the intervention: (1) ability to read and speak English; (2) ability to provide informed consent; (3) no history of falls within the past 12 months that resulted in hospitalization; (4) ability to walk 20 m without human assistance; (5) completion of the Timed Up and Go Test in less than 30 s [30]; (6) ability to read survey questions; and (7) completion of a post-consent comprehension test to ensure cognitive acuity for participation. The post-consent test asked participants to answer three questions regarding the study that were covered during the consent process. Specifically, participants were asked to: (1) Name two things he/she would be expected to do as a participant in the study; (2) Identify the duration of the study; and (3) Describe what he/she would do if he/she no longer wished to participate in the study. Because participants were enrolling into a research study, the post-consent test was used to ensure comprehension of study requirements (*i.e*., wearing the device belt, completing study surveys). The exclusion criteria required for safe participation in the intervention study, likely excluded older adults with functional impairment that may affect their mobility and the generalizability of these analyses. Further only those who could comprehend study procedures were included. This may have removed those with severe cognitive decline and also reduced our ability to detect associations with cognition. Only 8% of those screened were excluded due to a fall, and less than 2% due to cognitive problems or the timed up and go. All study activities took place at the retirement communities and participants were compensated $10 for the baseline measurement visit which lasted approximately 90 min. Ethical review and approval for the study was obtained from the University of California, San Diego Human Research Protections Program.

### 2.3. Measures and Procedures

#### 2.3.1. Demographics

Participants completed a self-report survey regarding demographic characteristics at the baseline measurement visit. Demographic variables included in the analyses were self-reported age, sex, and education. Education attainment was dichotomized (*i.e*., less than college *versus* college degree or above).

#### 2.3.2. Physical Functioning

To objectively measure physical functioning, participants completed the 400 Meter Walk Test (400MWT) [31] and Short Physical Performance Battery (SPPB) [32]. Participants were allowed to rest (without sitting down) during the 400MWT, but had to complete the task within 15 min. Individuals who did not complete the task in the allotted time were omitted from the analyses. The SPPB has been shown to predict disability [14,15] and assesses balance, strength (how long it takes to rise from a chair 5 times) and time to walk 4 m. We used the SPPB total score for the analyses. Pain interference was measured with the 6 item short form of the Patient-Reported Outcomes Measurement Information System (PROMIS) [33]. Standard procedures for PROMIS measures converted the raw scores into T-scores, with higher t-scores indicating more pain interference. See Table 1 for scoring protocols.

**Table 1:** Scoring protocol and descriptive statistics for dependent variables.

Variable	Scoring Protocol	Range	Sample Mean (SD)
Physical functioning			
Physical Performance Battery (SPPB)	Sum of scores on the three dimensions (*i.e*., balance, walk, chairs stands) ranging from 0 to 4	0–12	8.67 (2.74)
400 Meter Walk Test	Time in seconds to complete the 400 m	269–858	444.41 (114.04)
Pain Interference (PROMIS-PI)	T-score of sum across 6 five-level Likert items	41–64	49.72 (7.90)
Psychological functioning			
Fear of Falling (FES-I)	Sum across 16 five-level Likert items	16–64	25.79 (8.11)
Depression (CESD-10)	Sum across 10 four-level Likert items	0–18	5.51 (4.02)
Cognitive functioning			
Executive functioning, attention, visual search, and motor function (Trail Making Test)	Time in seconds to complete Trails B minus the time in seconds to complete Trails A	−13.09–275.78	90.25 (60.24)
Visual perception and information processing speed (Symbol search)	Number correct minus number incorrect within 2 min	1–47	19.86 (7.13)

#### 2.3.3. Psychological Functioning

To measure depressive symptoms, participants completed the 10-item Center for Epidemiological Studies Depression Scale (CES-D-10) [34,35]. Scores ranged from 0 to 30 with scores of 10 or higher indicating clinically significant depressive symptoms [34,35]. To measure fear of falling, participants completed the 16-item Falls Efficacy Scale International (FES-I) [36,37,38]. Scores on the FES-I ranged from 16 to 64 with scores of 23 or higher indicating a high concern for falling. See Table 1 for details.

#### 2.3.4. Cognitive Functioning

Participants completed the Trail Making Test A and B [39,40] and the Symbol Search subtest of the Wechsler Adult Intelligence Scale [41] to measure cognitive function (see Table 1). For the Trail Making Test, Trails A was completed first, followed by Trails B. Both items were scored using completion time in seconds and scores for participants who were unable to complete the test were set to the maximum value (180 s for Trails A and 300 s for Trails B). Trails A evaluates visual search and perceptual speed while Trails B examines working memory and task switching abilities [39,40]. Based on prior research, we estimated cognitive function by subtracting the completion time of Trails A from Trails B (Trails B time–Trails A time) [42]. The Symbol Search assesses visual perception and information processing speed by presenting participants with two symbols to the left of a set of five symbols aligned in a row [41]. Participants were given 2 min to determine row by row if the set of five symbols on the right included either of the two symbols on the left. A summary score was calculated by subtracting the number incorrect from the number correct within the 2-min testing period.

#### 2.3.5. GPS and Accelerometer

For the baseline measurement, participants were asked to wear a BT1000X GPS tracking device (Qstartz International Co. Ltd, Tapei, Taiwan) which has an accuracy of 3 m [43]. Participants were instructed by study staff how to charge the device every evening to ensure compliance. To measure physical activity, participants wore an Actigraph GT3X+ accelerometer (Actigraph, Inc., Pensacola, FL, USA), which was affixed to the same belt as the GPS device. Participants were asked to wear the belt for 6 days for a minimum of 10 h per day and were asked to re-wear the belt if these criteria were not met on at least 4 days based on screening of the accelerometer data. The standard for measuring physical activity via accelerometer typically requires a 7 day wear period [44] with five valid days accepted for data analysis; however, because the MIPARC population involved a sample of older adults who were retired or non-working, the need to include weekend days was not as important because the patterns of behavior in this population has been shown to be consistent across days [45]. The device deployment was conducted at the sites and on the same day each week to help participants, so only 6 days of wear were available. Because the accelerometer and GPS devices were affixed to the same belt, the same 5 day valid wear period was applied to the GPS data.

### 2.4. Data Processing

Following the wear period, data from the GPS were merged to the accelerometers by time stamp using the Personal Activity Location Measurement System (PALMS) software. PALMS is a web-accessible service that is used to store, process, and merge time-stamped data from these types of devices [22,46]. PALMS aggregated these epoch-based data at the 1-minute level (*i.e*., each row of data represented 1 person-minute), with one accelerometer value (*i.e.,* count per minute) and one GPS coordinate noted for each minute. PALMS was used to remove potential errors in latitude and longitude coordinates caused by indoor jitter (*i.e*., indoor GPS signal interference) and outdoor multipath reflections (*i.e*., GPS signal interference caused by large urban canyons or high density canopies) [46]. PALMS was used to remove non-wear time based on the accelerometer. Non-wear time was defined as 90 consecutive minutes of zeros with a two minute threshold [47]. Days when the accelerometer was not worn for at least 10 h were excluded from the analyses. Because the GPS device is affixed to the same belt as the accelerometer and is difficult to remove from the belt, the same wear criteria for the accelerometer was applied to the GPS data.

For the purposes of this paper, PALMS was used to identify potential trips and transportation mode. Trips were defined as groups of sequential GPS coordinates (≥2 fixes) of latitude and longitude coordinates that spanned ≥25 m with an average speed across fixes of ≥1.5 km/h [22]. We chose a short minimum trip distance of 25 m and trip time of 2 min due to the physical limitations of this population. PALMS does not require the origin and destination of a trip to be a different location; therefore, it is unknown whether or not a trip is for transportation or leisure. To classify transportation mode, trips with a 90th percentile speed of ≥10 km/h were classified as vehicle trips and trips with a 90th percentile speed of <10 km/h were classified as walking trips. Because of the potential of misclassification of bicycling with this method, and the very small proportion of participants reporting bicycling, we eliminated participants who self-reported bicycling (N = 5). We also performed additional processing to maximize trip detection and mode classification accuracy with techniques that have been validated elsewhere [22]. Unfortunately PALMS does not employ the accelerometer data in its transportation algorithms and we have shown that additional accelerometer criteria improve predictions [22]. The following additional criteria were applied: (1) vehicle trips that were falsely identified as trips due to remaining GPS scatter (un-detected by PALMS) were removed if the probability of scatter was greater than 60% based on a validated algorithm [22]; (2) walking trips that were falsely identified as trips due to scatter (*i.e*., probability greater than 60%) were removed if the accelerometer showed a mean CPM of less than 500 during the trip (walking CPM was based on counts seen in 400 m walking tests in this population); and (3) any walking trips with a mean CPM of less than 250 were also removed (walking CPM was based on counts seen in 400 m walking tests in this population). These decisions improved the valid trip detection by 30% for the walking trips and 28% for the vehicle trips.

#### Data Aggregation

The aforementioned minute-level data were aggregated using SPSS 21 to create trip-, day-, and participant-level datasets which were used to address the study aims. Aggregation proceeded in the following three steps:

First, the data were aggregated to the trip level, where each case represented a unique participant trip (N = 6263 trips). Summary variables were created for each trip, including trip time (in minutes), distance (in kilometers), and mode (pedestrian or vehicle). Additionally, a physical activity summary of mean *counts per minute* (CPM) from accelerometer data was created for each trip.

Secondly, this trip level file was aggregated to create a day-level dataset which summarized daily trip characteristics for each participant day (N = 1675 participant days). Summary variables included total daily number of trips, minutes traveled, and distance traveled, stratified by transportation mode.

Thirdly, the day-level file was aggregated to create a participant-level dataset which summarized trip characteristics for each participant across all of their days of wearing the devices (N = 279 participants). Summary variables were (1) mean number of trips per day; (2) mean distance traveled per day; and (3) mean minutes traveled per day, calculated separately for pedestrian and vehicle trips.

### 2.5. Analyses

Mean and standard deviation number of trips per day, distance traveled per day, and daily minutes for pedestrian and vehicle trips were calculated across all valid days of monitoring using the day-level data. Using the participant-level data, separate linear mixed-effects multilevel regression models were used to evaluate the relationship between travel behaviors (mean daily number of trips, distance traveled, and daily minutes for pedestrian and vehicle trips) and each of the individual health characteristics; resulting in 6 independent variables and 7 dependent variables. Site was included as a random effect to account for clustering by retirement community. Covariates included age, gender, and educational status. To eliminate any potential bias based on participants wearing the accelerometer and GPS devices for varying lengths of time, mean daily accelerometer wear-time was included in all models. Participants with one or more missing item on a scale were classified as missing on the scale to preserve psychometric properties. Standardized regression coefficients (β) were calculated using z-scores for the independent and dependent variables so associations could be compared across models, and are reported in addition to unstandardized regression coefficients (B). All data processing and analyses were carried out using SPSS v22 software with significance set at *p* < 0.05.

## 3. Results and Discussion

### 3.1. Results

A total of 279 (range 253– 279) participants were included in the analyses. Participant characteristics are described in Table 2. On average, participants were 83 ± 6 years old and 71% were female. Participants wore the accelerometer an average of 13.6 h per day (1.3 SD) for a mean of 5.1 days (1.9 SD). In the day-level data, participants had a mean of 10 min (10.6 SD) in pedestrian trips and 13.2 min (14.6 SD) in vehicle trips. The mean daily distance traveled by pedestrian trip was 0.5 km (0.6 SD) compared to a mean of 8.7 km (18.8 SD) for vehicle trips. Average accelerometer counts (that represent intensity of movement) during pedestrian trips were 1188.8 CPM (714.4 SD) and 219.7 CPM (317.1 SD) for vehicle trips. See Table 3 for additional details.

**Table 2:** Demographics of participants included in the analyses.

Demographic	Mean (SD)/Frequency (%)
Age	83 (6.3)
Gender	
Men	81 (28.7)
Women	201 (71.3)
Education	
College and above	180 (64.7)
Below college	98 (35.3)
Wear-time (hours/day)	13.6 (1.3)

**Table 3:** Day-level descriptives across all wear days.

GPS Trip Variables	Pedestrian [Mean (SD)]	Vehicle [Mean (SD)]
Daily time in trips based on GPS data (minutes)	10.2 (10.6)	13.2 (14.6)
Daily distance in trips based on GPS data (km)	0.5 (0.6)	8.7 (18.8)
Daily activity during trips based on accelerometer data (cpm)	1188.8 (714.4)	219.7 (317.1)

#### 3.1.1. Pedestrian Travel

There was a significant positive relationship between the mean daily number, distance, and minutes of pedestrian trips with the SPPB indicating that participants who on average had more pedestrian trips, and traveled further and spent more time in pedestrian trips had higher scores on the SPPB (see Table 4). A significant negative association was found with mean daily number, distance, and minutes of pedestrian trips to the 400 Meter Walk Time indicating that those participants with more trips, with a further distance and more minutes in the behavior completed the walking task faster. There was a significant negative association with mean daily number, distance, and minutes of pedestrian trips to self-reported pain interference. There were significant negative associations with mean daily number, distance, and minutes of pedestrian trips to fear of falls and depressive symptoms. Participants who had more pedestrian trips and traveled further with more time, reported lower fear of falling and fewer depressive symptoms. There were no significant associations for pedestrian trip variables (*i.e*., number, distance, minutes) with Trails A or B or the Symbol Search (see Table 4).

**Table 4:** Relationship between number, distance, and minutes of pedestrian trips with health characteristics.

	Mean Daily Number of Pedestrian Trips		Mean Daily Distance Traveled in Pedestrian Trips (per 10 m)		Mean Daily Time Traveled in Pedestrian Trips (per 10 min)	
	B (95% CI)	β	B (95% CI)	β	B (95% CI)	β
**Physical functioning**						
Physical Performance Battery (SPPB)	0.46 (0.20, 0.72) *	0.22	0.01 (0.00, 0.10) **	0.23	0.35 (0.20, 0.50) **	0.22
400 Meter Walk ª	−40.57 (−51.58, −29.57) **	−0.47	−0.44 (−0.60, −0.30) **	−0.37	−24.47 (−32.10, −1.69) **	−0.40
Pain Interference ª (PROMIS-PI)	−1.29 (−2.14, −0.44) *	−0.26	−0.02 (−0.03, −0.01) **	−0.21	−1.18 (−1.8, −0.60) **	−0.26
**Psychological functioning**						
Fear of Falling ª (FES-I)	−1.08 (−1.90, −0.26) *	−0.17	−0.02 (−0.03, −0.01) *	−0.18	−0.80 (−1.40, −0.20) *	−0.17
Depression ª (CESD-10)	−0.49 (−0.92, −0.06) *	−0.16	−0.01 (−0.01, −0.00) *	−0.20	−0.47 (−0.80, −0.20)	−0.20
**Cognitive functioning**						
Trail Making Test ª	−0.46 (−6.25, 5.30)	−0.01	−0.01 (−0.01, 0.01)	−0.01	−0.04 (−4.10, 4.10)	−0.00
Symbol search	0.15 (−0.57, 0.88)	0.03	0 (−0.01, 0.01)	0.01	0.04 (−0.50, 0.60)	0.01

Notes: * = significant at α < 0.05; ** = significant at α < 0.001. ª Negative associations indicate higher functioning and less impairment based on the measures. B = unstandardized regression coefficient; β = standardized regression coefficient (indicates a one standard deviation change in IV and DV).

#### 3.1.2. Vehicle Transportation

There were no significant associations for the vehicle trip variables (*i.e*., number/day, distance/day, minutes/day) with the SPPB, 400 m walk, or pain interference. There was a significant negative relationship between mean daily number of vehicle trips and fear of falling, indicating that those individuals who had more daily vehicle trips had a lower fear of falling. There were no significant associations for mean daily distance and minutes of vehicle trips with fear of falling or mean daily number, distance, or minutes of vehicle trips with depressive symptoms. Additionally, there were no significant associations for vehicle trip variables (*i.e*., number, distance, minutes) with Trails A or B or the Symbol Search (see Table 5).

**Table 5:** Relationship between number, distance, and minutes of vehicle trips with health characteristics.

	Mean Daily Number of Vehicle Trips		Mean Daily Distance Traveled in Vehicle Trips (per 10 km)		Mean Daily Time Traveled in Vehicle Trips (per 10 min)	
	B (95% CI)	β	B (95% CI)	β	B (95% CI)	β
**Physical functioning**						
Physical Performance Battery (SPPB)	0.20 (−0.05, 0.45)	0.09	0.04 (−0.10, 0.10)	0.04	0.09 (−0.03, 0.20)	0.08
400 Meter Walk ª	−8.74 (−20.28, 2.80)	−0.09	−0.30 (−5.0, 4.40)	−0.01	−2.01 (−7.30, 3.20)	−0.05
Pain Interference ª (PROMIS-PI)	−0.11 (−0.95, 0.74)	−0.02	−0.13 (−0.50, 0.20)	−0.05	−0.13 (−0.50, 0.30)	−0.04
**Psychological functioning**						
Fear of Falling ª (FES-I)	−0.89 (−1.70, −0.09) *	−0.13	−0.05 (−0.40, 0.30)	−0.02	−0.22 (−0.60, 0.10)	−0.07
Depression ª (CESD-10)	−0.23 (−0.65, 0.19)	−0.07	−0.04 (−0.20, 0.10)	−0.03	−0.06 (−0.30, 0.10)	−0.04
**Cognitive functioning**						
Trail Making Test ª	0.43 (−5.27, 6.12)	0.01	−1.61 (−4.0, 0.70)	−0.08	−1.45 (−4.1, 1.10)	−0.06
Symbol search	0.36 (−0.35, 1.07)	0.06	0.12 (−0.20, 0.40)	0.05	0.22 (−0.10, 0.50)	0.08

Notes: * = significant at α < 0.05; ** = significant at α < 0.001. ª Negative associations indicate higher functioning and less impairment based on the measures. B = unstandardized regression coefficient; β = standardized regression coefficient (indicates a one standard deviation change in IV and DV).

### 3.2. Discussion

This study is among the first to assess transportation mode objectively with GPS devices and relate this to important multiple health characteristics in older adults. Greater life-space mobility (*i.e*., travelling further in the community) which might be achieved through vehicle travel, did not appear to be related to functioning in older adults in retirement communities. Fear of falls was the only health characteristic related to number of vehicle trips. Physical mobility, as measured by walking trips, had moderate associations with multiple physical and psychological functioning indicators. It is possible that providing supportive environments and programs that support walking could benefit multiple aspects of health in older adults. Furthermore, the association between walking and health, particularly functioning, is likely cyclical, so supporting improved functioning through multiple mechanisms (e.g., walking, muscle strengthening) is likely needed to maximize health.

There was no association between pedestrian trips and cognitive functioning. However, education status has been shown to be highly correlated with cognitive ability [48] which may limit our ability to identify such a relationship in this highly educated cohort. There were significant associations with pedestrian transportation (*i.e*., number, distance, minutes) and physical functioning which may be a result of the physical activity involved in walking. This association was also likely due to individuals with better functioning being physically able to walk more, though our inclusion criteria resulted in eliminating those with severe physical impairments. There was also a strong negative association with greater pedestrian transportation and less fear of falls and depression. Previous research has highlighted the physical benefits of walking in older adult populations and the results from this study used a new measurement method with evidence of validity to further explore the relationship between physical health and specific metrics of walking trips. All three metrics, number of trips, distance and minutes were associated with trip variables. While longer trips would be expected to be related to health, a greater number of trips, which could also include short trips, was also important.

No significant associations were found with vehicle transportation and physical and cognitive functioning. There was a small and significant negative association between the number of vehicle trips and fear of falling, but not vehicle distance or minutes traveled. Those who had more vehicle trips had less fear of falling. Fear of falling has been previously associated with walking but not vehicle travel. This indicates that falls may affect life-space mobility as well as physical mobility. Avoiding falls through safe environments, medication surveillance and balance training should be a priority for older adults [49,50]. The lack of an association between vehicle transportation and cognitive functioning could have been due to the fact that GPS cannot determine if the participant was the driver or passenger of the vehicle, which may require different cognitive abilities. Future research could further elucidate this relationship with the use of person worn cameras in combination with GPS data to determine participants’ driving behavior [18,19].

Our findings differ from some previous studies using GPS. For example, Wettstein *et al.* found that cognitive status did affect the complexity of mobility activities [26]. In addition self-reported Life-Space has been associated with cognitive impairments [51,52]. These studies, however, likely included older adults with greater cognitive decline than participants in our study.

### 3.3. Strengths and Limitations

This study was among the first to assess the relationship between GPS measured transportation behavior and physical, cognitive and psychological functioning in older adults living in retirement communities. Our study sample, while very old (average 84 years) were functionally mobile based on falls and timed up and go assessments required for participants in the study intervention and cognitive capable of participating in complex study. Our findings may not generalize to a more mobile population or those with severe cognitive impairments. While we employed validated algorithms in a two-step process, some error in GPS trip classification may still occur. Our study is strengthened by the inclusion of accelerometer data and counts derived from observed walking tests in this population and person worn camera data of acceleration observed in vehicle trips [22]. It is also possible that some trips were missed when participants were not wearing the study belt. However, our ability to assess whether the GPS was worn is much improved by the validated wear time criterion for the accelerometer which was on the same belt as the GPS. The cross-sectional data from this study do not shed light on the causal relationship between transportation behaviors and health characteristics. It is likely that the relationships are bi-directional and this could be assessed in future studies using longitudinal designs. For example, participants with higher physical and psychological functioning may be better able to engage in pedestrian travel or those with more pedestrian travel may have better functioning. Therefore, supporting functional and cognitive health in older adults likely requires strategies to support walking (e.g., improving access to walkable environments) as well as strategies (e.g., correct medication, vision and hearing testing *etc.*) that support health across all domains (*i.e*., physical, psychological, and cognitive functioning).

## 4. Conclusions

Significant relationships were found with pedestrian transportation behaviors and health characteristics. These significant relationships may be due to the increased physical activity associated with walking transportation and the cyclical link between physical functioning and walking mobility. Increasing opportunities for walking and improved functioning in older adult populations may be especially valuable for health. This study used novel GPS techniques to evaluate these relationships and demonstrated the utility and construct validity of GPS data to explore transportation behaviors.
